# Deep learning-based survival prediction for multiple cancer types using histopathology images

**DOI:** 10.1371/journal.pone.0233678

**Published:** 2020-06-17

**Authors:** Ellery Wulczyn, David F. Steiner, Zhaoyang Xu, Apaar Sadhwani, Hongwu Wang, Isabelle Flament-Auvigne, Craig H. Mermel, Po-Hsuan Cameron Chen, Yun Liu, Martin C. Stumpe

**Affiliations:** 1 Google Health, Google LLC, Palo Alto, California, United States of America; 2 Advanced Clinical, Chicago, Illinois, United States of America; Chang Gung Memorial Hospital at Linkou, TAIWAN

## Abstract

Providing prognostic information at the time of cancer diagnosis has important implications for treatment and monitoring. Although cancer staging, histopathological assessment, molecular features, and clinical variables can provide useful prognostic insights, improving risk stratification remains an active research area. We developed a deep learning system (DLS) to predict disease specific survival across 10 cancer types from The Cancer Genome Atlas (TCGA). We used a weakly-supervised approach without pixel-level annotations, and tested three different survival loss functions. The DLS was developed using 9,086 slides from 3,664 cases and evaluated using 3,009 slides from 1,216 cases. In multivariable Cox regression analysis of the combined cohort including all 10 cancers, the DLS was significantly associated with disease specific survival (hazard ratio of 1.58, 95% CI 1.28–1.70, p<0.0001) after adjusting for cancer type, stage, age, and sex. In a per-cancer adjusted subanalysis, the DLS remained a significant predictor of survival in 5 of 10 cancer types. Compared to a baseline model including stage, age, and sex, the c-index of the model demonstrated an absolute 3.7% improvement (95% CI 1.0–6.5) in the combined cohort. Additionally, our models stratified patients within individual cancer stages, particularly stage II (p = 0.025) and stage III (p<0.001). By developing and evaluating prognostic models across multiple cancer types, this work represents one of the most comprehensive studies exploring the direct prediction of clinical outcomes using deep learning and histopathology images. Our analysis demonstrates the potential for this approach to provide significant prognostic information in multiple cancer types, and even within specific pathologic stages. However, given the relatively small number of cases and observed clinical events for a deep learning task of this type, we observed wide confidence intervals for model performance, thus highlighting that future work will benefit from larger datasets assembled for the purposes for survival modeling.

## Introduction

The ability to provide prognostic information in oncology can significantly impact clinical management decisions such as treatment and monitoring. One of the most common systems for this purpose is the American Joint Committee on Cancer (AJCC) “TNM” cancer staging system, whereby tumors are classified by primary tumor size/extent (T), lymph node involvement (N), and the presence or absence of distant metastasis (M). Although TNM staging is useful and well-studied, there is room for improvement in some settings, with ongoing efforts to develop improved prediction strategies that incorporate information such as clinical variables [1,2], genetic information [3,4], and histomorphological features including tumor grade [5]. In this regard, computational image analysis of tumor histopathology offers an emerging approach to further improve patient outcome predictions by learning complex and potentially novel tumor features associated with patient survival.

In recent years, deep learning has been shown to recognize objects [6] and diagnose diseases from medical images [7,8] with impressive accuracy. In pathology, prior studies have reported deep learning models with performance on par with human experts for diagnostic tasks such as tumor detection and histologic grading [8–10]. The main advantage of deep learning-based approaches relative to prior approaches based on manually engineered features (e.g. nuclear size/shape), is the automated learning of prognostic features, without the need for prior assumptions or dependence on a limited set of known features. One downside of deep learning however is that it generally requires large annotated datasets to work well. In histopathology, these models are typically trained on millions of small image patches taken from whole-slide images (WSIs) of digitized pathology slides that have had specific features of interest labeled by pathologists, often involving detailed, hand-drawn annotations. The reliance on expert annotation has two notable disadvantages. Firstly, these annotations are laborious for experts, requiring hundreds to thousands of hours per prediction task of interest and limiting the ability to quickly extend to new applications such as other cancer types or histologic features. Examples of such annotations include outlines of the locations of metastatic tumor, or labeling every region (e.g. gland) in the sample with its corresponding tumor grade [8–10]. These annotated regions can then be used to generate example image patches of each category of interest. Secondly, the annotations explicitly enforce that the learned morphologic features are correlated with the known patterns being annotated. This may be especially difficult if the prognostic label is currently unknown, or if the desire is to learn novel prognostic features.

By contrast, a different line of work focuses on directly learning morphologic features associated with survival without reliance on expert annotation for known pathologic features or regions of interest. Such approaches instead provide the machine learning models with a single “global” label per slide or case, such as a specimen’s mutational status or a patient’s clinical outcome. The task of predicting clinical outcomes from WSIs is particularly challenging due to the large size of these images (approximately 100,000×100,000 pixels at full resolution) along with the notion that the morphologic features associated with survival may, in principle, appear in any part of the imaged tissue. The large amount of image data in conjunction with morphological heterogeneity and unknown discriminative patterns result in an especially challenging, “weakly-supervised” learning problem.

Several prior efforts using machine learning and WSIs to address the survival prediction problem have used data from The Cancer Genome Atlas (TCGA), the largest publicly available database to our knowledge of digitized WSIs paired with clinical and molecular information [11–17]. These prior works have used feature-engineering approaches [13,16], leveraged annotated regions of interest [12,18,19], focused on learning of known histologic features [17] and/or developed models to directly predict survival for an individual cancer type. Here, we build on and extend prior work by developing an end-to-end deep learning system (DLS) to directly predict patient survival in multiple cancer types, training on whole-slide histopathology images without leveraging expert annotations or known features of interest. We test several loss functions to address the problem of right-censored patient outcomes, a convolutional neural network that is directly optimized to extract prognostic features from raw image data, and an image subsampling method to tackle the large image problem.

We evaluated our DLS’s ability to improve risk stratification relative to the baseline information of TNM stage, age, and sex for 10 cancer types from TCGA. Though we observed improved risk stratification based on the model predictions for several cancer types, effect sizes were difficult to estimate precisely due to the limited number of cases and clinical events present in TCGA (350–1000 cases and 60–300 events per cancer type). While the results reported here provide support for the feasibility of developing weakly supervised deep learning models to predict patient prognosis from whole-slide images across multiple cancer types, future work exploring and validating the potential of deep learning applications for this task will require larger, clinically representative datasets.

## Materials and methods

### Data

Digitized whole-slide images of hematoxylin-and-eosin- (H&E-) stained specimens were obtained from TCGA [20] and accessed via the Genomic Data Commons Data Portal (https://gdc.cancer.gov). Images from both diagnostic formalin-fixed paraffin-embedded (FFPE) slides and frozen specimens were included. Based on initial experiments as well as differences in the proportion of available FFPE images across cancer types (i.e. TCGA studies), both the FFPE and frozen WSIs available for each patient were used for training and case-level predictions. Each case contained 1–10 slides (median: 2). Clinical data (including approximated disease specific survival) were obtained from the TCGA Pan-Cancer Clinical Data Resource [21] and the Genomic Data Commons.

Of the TCGA studies for which cancer stage data were available, we chose the 10 studies with the highest number of cases and survival events. Clinical stage was used only for ovarian serous cystadenocarcinoma (OV), which did not have pathologic stage data available but was included given the high number of observed events. Cutaneous melanoma (SKCM) was excluded as it was not restricted to primary, untreated tumors [14,22]. Thyroid carcinoma (THCA) was excluded because only 14 of 479 cases had an observed event. Cases with missing data for either pathologic stage, age, sex, or disease specific survival were excluded from evaluation, whereas only cases missing disease specific survival were excluded from model development (training and tuning).

For each TCGA study, cases were split into train, tune, and test sets in a 2:1:1 ratio. To ensure representative splits given the small number of cases, split assignment was further stratified on whether the time of disease specific survival event was observed, and the time-to-event (discretized into 3 intervals based on the 25^th^ and 75^th^ percentiles). Across all cancer types, 4,880 cases (12,095 images) were used for training and tuning. The remaining 1,216 cases (3,009 images) were used for evaluation (Table 1). The pathologic stage distribution for each TCGA study and split is detailed in S1 Table.

**Table 1:** Dataset overview.

Study	Cases			DSS Events (%)			Slides		
	Train	Tune	Test	Train	Tune	Test	Train	Tune	Test
**BLCA (bladder urothelial carcinoma)**	197	98	96	62 (31%)	31 (32%)	30 (31%)	437	205	206
**BRCA (breast invasive carcinoma)**	488	247	250	40 (8%)	19 (8%)	20 (8%)	1182	599	631
**COAD (colon adenocarcinoma)**	218	110	103	32 (15%)	16 (15%)	13 (13%)	625	313	310
**HNSC (head and neck squamous cell carcinoma)**	196	99	101	52 (27%)	27 (27%)	28 (28%)	481	250	247
**KIRC (kidney renal clear cell carcinoma)**	260	130	130	55 (21%)	27 (21%)	27 (21%)	777	395	382
**LIHC (liver hepatocellular carcinoma)**	165	83	85	32 (19%)	17 (20%)	18 (21%)	341	165	172
**LUAD (lung adenocarcinoma)**	233	115	112	54 (23%)	28 (24%)	26 (23%)	619	283	282
**LUSC (lung squamous cell carcinoma)**	219	108	109	45 (21%)	22 (20%)	21 (19%)	542	275	269
**OV (ovarian serous cystadenocarcinoma)**	272	133	137	151 (56%)	73 (55%)	76 (55%)	607	299	298
**STAD (stomach adenocarcinoma)**	198	95	93	48 (24%)	24 (25%)	25 (27%)	464	227	212
**Combined**	2446	1218	1216	571 (23%)	284 (23%)	284 (23%)	6075	3011	3009

Our datasets were derived from The Cancer Genome Atlas (TCGA). Cases with known disease specific survival (DSS), pathologic stage, age, and sex were assigned into train, tune, and test splits in a ratio of 2:1:1. Each TCGA study code refers to a cancer type, and “Combined” refers to all 10 studies combined. Cancer stage distribution is presented in S1 Table.

### Deep Learning System (DLS)

#### Neural network architecture

**Fig 1:**
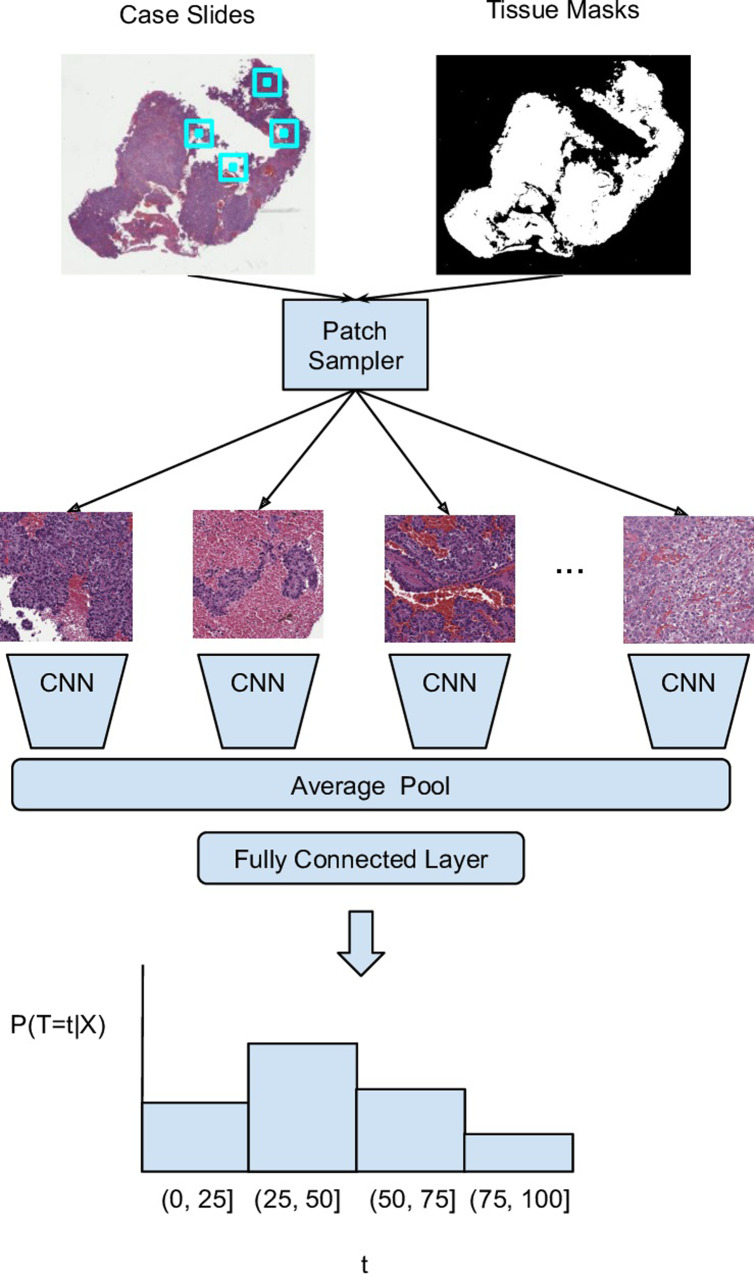
Summary of the weakly supervised learning approach for directly predicting disease specific survival. For each case, cropped image patches were uniformly sampled from tissue-containing areas across all slides available for a given case. Next, image features were extracted for each patch by a convolutional neural network (CNN). These patch-level features were averaged (on a per-channel basis) and fed to a fully connected layer. Our custom loss function divided the follow-up period into four discrete bins depending on right-censorship time and outcome (Methods). As such, the model was designed to output a probability distribution over discretized survival times.

The core element of our deep learning system (DLS) consisted of multiple convolutional neural network (CNN) modules with shared weights, and an average pooling layer that merges image features computed by these modules (Fig 1). Our CNN consisted of layers of depth-wise separable convolution layers, similar to the MobileNet [23] CNN architecture. The layer sizes and the number of layers were tuned for each study via a random grid-search (see S2 Table and S1 Algorithm). We chose this family of architectures because they contain relatively few parameters compared to other modern CNN architectures, which speeds up training and helps to reduce the risk of overfitting. Each CNN module took as input a randomly selected image patch from the slides in each case, such that when multiple patches were sampled, probabilistically at least one patch was likely to be informative of the outcome. Specifically, if the frequency of informative patches on a slide is *p*, the probability of not sampling any informative patch in *n* patches decays exponentially with *n*: (1-*p*)^*n*^, shrinking towards zero with even moderate values of *n*. This approach thus handles the weak label nature of survival prediction on large images, where the location of the informative region in the image or set of images is unknown. Furthermore, this approach naturally generalizes to multiple slides per case. During each training iteration, the n patches were sampled randomly, further ensuring that informative patches were sampled across training iterations.

Each patch was of size 256 pixels and was sampled uniformly at random from tissue-containing regions within all slides in a case. Early experiments with different patch sizes did not yield meaningful improvements. The CNN then extracted image-based features from the patches. A top-level average-pooling layer allowed the model to take as input different numbers of patches between training and evaluation. This enabled the use of a smaller number of patches and resultant higher case diversity during training, and a more extensive coverage of slides in each case with a larger number of patches during evaluation. A final logistic regression layer produced a prediction given the output of the average pooling layer.

#### Survival loss functions

We experimented with three different loss functions for training the DLS. Early experiments (evaluated on the tune split) showed that the Censored Cross-Entropy described below gave the best results (S1 Fig) and was used for final model training.

The first tested loss function was based on the Cox partial likelihood [24], which is used for fitting Cox proportional hazard models, but can be extended to train neural networks as follows:

$$\max\underset{i:O_{i}=1}{\prod }\frac{e^{f\left(X_{i}\right)}}{\sum_{j:T_{j}\geq T_{i}}e^{f\left(X_{j}\right)}}$$

Where *T*_*i*_ is the event time or time of last follow-up, *O*_*i*_ is an indicator variable for whether the event is observed, *X*_*i*_ is the set of whole slide images and *f(X*_*i*_*)* is the DLS risk score, each for the *i*^th^ case. In our implementation, we used Breslow's approximation [25] for handling tied event times. In principle, the loss for every single example is a function of all cases in the training data. In practice, we approximated the loss at each optimization step, by evaluating it over the examples in a small batch (n ≤ 128) instead of the entire training dataset.

Our second loss function was an exponential lower bound on the concordance index [26]. The concordance index is a common performance metric for survival models that corresponds to the probability that a randomly chosen pair of subjects is correctly ordered by the model in terms of event times. The concordance index itself is not differentiable, however, Raykar et al. [27] proposed the following differential lower bound that can be used for model optimization:

$$E\;:=\left\{\left(i,j\right)|O_{i}=1\;and\;T_{j}>T_{i}\right\}$$

$$\max\underset{\left(i,j\right)\in E}{\sum }1-e^{f\left(X_{i}\right)-f\left(X_{j}\right)}$$

Where *E* is the set of pairs of examples (*i*, *j*) where the *i*^th^ event is observed and *T*_*j*_*>T*_*i*_. Similar to the Cox partial likelihood, we approximated this lower bound on the concordance index at each optimization step by evaluating it over the examples in a small batch (n ≤ 128) instead of the entire training dataset.

The final loss function, the censored cross-entropy, is an extension of the standard cross-entropy loss used for classification models to train survival prediction models with right-censored data. We modeled survival prediction as a classification problem instead of a regression or ranking problem, by discretizing time into intervals and training models to predict the discrete time interval in which the event occurred instead of a continuous event time or risk score. For examples with observed events, the standard cross-entropy was computed. However for censored examples, the time interval in which the event occurs is unknown. Therefore, we leverage the information that the event did not occur before the censorship time and maximize the log-likelihood of the event occurring in the interval of censorship or thereafter. The full loss function can be written as follows:

$$max\underset{i}{\sum }\left[O_{i}*\log\left(f\left(X_{i}\right)\left[Y_{i}\right]\right)+\left(1-O_{i}\right)*\text{log}\left(\underset{y>Z_{i}}{\sum }f\left(X_{i}\right)\left[y\right]\right)\right]$$

Where *Y*_*i*_ is the interval in which the event occurred (for example with observed events) and *Z*_*i*_ is the latest interval whose endpoint is before the time of censorship (for censored examples). *f(x)* is a predicted probability distribution over time intervals and *f(x)[y]* is the probability assigned by the model for the event occurring in the *y*^*th*^ interval. An important design consideration when using this loss function is how to discretize time. We used different percentiles of the time to death distribution for non-censored cases (i.e. quartiles). Discretization was done separately for each study to account for the considerable differences in survival times across studies (cancer types). To obtain a scalar risk score for evaluation, we took the negative of the expectation over the predicted time interval likelihood distribution. The negation ensured that higher risk score values indicate higher risk.

#### Training procedure

Training examples consisted of sets of up to 16 image patches per case sampled uniformly from tissue across all the slides in that case. Tissue detection using a pixel-intensity-based threshold as well as data augmentation via stain normalization followed by color and orientation perturbations were both performed as described previously [9]. Training was performed using WSIs for both frozen and FFPE specimens. Numerical optimization of network parameters was done using the RMSProp optimizer [28] in TensorFlow in a distributed fashion, using 10 worker machines with 16 processors each.

For each study, we tuned the hyperparameters by randomly sampling 50 hyperparameter configurations and then training one model with each configuration for each of the 10 studies (500 models in total). The hyperparameter search space is detailed in S2 Table.

#### Evaluation procedure

At evaluation we sampled 1024 patches per case, using the same procedure as during training. Early empirical studies using the tune set showed no performance benefit from sampling more patches. The final models used for evaluation were averaged in a number of ways. First, model weights were the exponential moving average of model weights across training steps, with a decay constant of 0.999. Next, instead of picking a single best training checkpoint (i.e. a model evaluated at a particular training step) for each study, we used an ensemble of 50 checkpoints. Each model was trained for 500,000 steps and evaluated every 25,000 training steps, yielding 20 checkpoints per model, and a total of 1,000 checkpoints across 50 hyperparameter settings. The 50 checkpoints that achieved the highest c-index on the tune set were selected for the ensemble. The final ensemble prediction was the median of the 50 individual predictions.

### Survival analysis

To avoid clinically irrelevant comparisons (e.g. 107 days vs 108 days), survival times were discretized from days to months for all analyses. For the Kaplan-Meier analysis, cases were first stratified into risk groups within each cancer type by choosing different risk score quantiles as thresholds. Stratification per cancer type is important because it ensures that the distribution of cancer types is the same across all risk groups. Without doing this it would have been possible to see differences in risk groups simply because one risk group contains more cases from cancers with a worse prognosis (e.g. OV vs BRCA). For the KM analysis by stage, we repeated the same procedure for cases from each stage.

We used Cox proportional hazards regression [29] as both an analytical tool and a predictive model. We used it first as an analytical tool for determining which variables were correlated with disease-specific survival by fitting multivariable models that include the DLS risk scores and baseline variables to our test dataset. The pathologic stage was encoded as a numeric variable (i.e. 1, 2, 3, 4) in this analysis, because there were insufficient data for using dummy variables for many studies. Age was also treated as a numeric variable. Age was divided by 10, so that the hazard ratio corresponds to the increased risk from an additional 10 years of age at the time of diagnosis. We fit a separate model for each study and a model across all studies combined. For the combined model, a dummy indicator variable for the cancer type was added.

In the second analysis, where we examined the additional prognostic value of adding the DLS to a multivariable model, we needed to control for the natural improvements in model fit with more input variables. Thus we used Cox regression as a predictive model, in conjunction with leave-one-out cross validation (LOO) across test set cases. In this analysis, prognosis prediction performance was measured using the c-index, an extension of the AUC for binary outcomes without censorship [30]. Briefly, the concordance (“c”) index is the number of “concordant” pairs (cases that were correctly ordered given the outcome and censorship time) divided by all informative pairs. Because different studies (cancer types) had markedly different followup periods and median survival times, the c-indices for the “combined” study summed concordant pairs and informative pairs solely within the same study. For example, the concordance index for the combined studies A and B was calculated as (concordant-pairs_A_ + concordant-pairs_B_) / (informative-pairs_A_ + informative-pairs_B_).

### Statistical analysis

Survival analysis was conducted using the Lifelines python package (version 0.24.0) [31]. For Kaplan Meier analysis, risk groups were determined based on the quantile: upper and lower quartiles were defined to be high-risk and low-risk groups, while the remaining were defined as medium risk. Survival curves for the high-risk and low-risk groups were compared using the log-rank test (“statistics.logrank_test”). Cox proportional hazards regression analysis [29] (including confidence intervals and p-values) were conducted by fitting the continuous (non-binarized) predictions of the DLS using the “CoxPHFitter” function. The c-index was computed using the “utils.concordance_index” function. Confidence intervals for the c-index and the delta in c-index between models were generated using the non-parametric bootstrap approach (sampling with replacement) with 9,999 samples.

### Heatmap analysis

Risk heatmaps for patch analysis were generated by running the DLS on a single patch at a time to produce patch-level DLS risk scores across entire slides. To generate visualizations for pathologist review, patches were sampled based on patch-level risk score from the top 25% and bottom 25% from each case. Patches were grouped by case and cases were organized by patient-level risk prediction. These organized patches were then reviewed by two pathologists to qualitatively evaluate high-level features that may be associated with both the case-level and patch-level risk scores.

## Results and discussion

### Comparing survival rates in low and high risk groups

**Fig 2:**
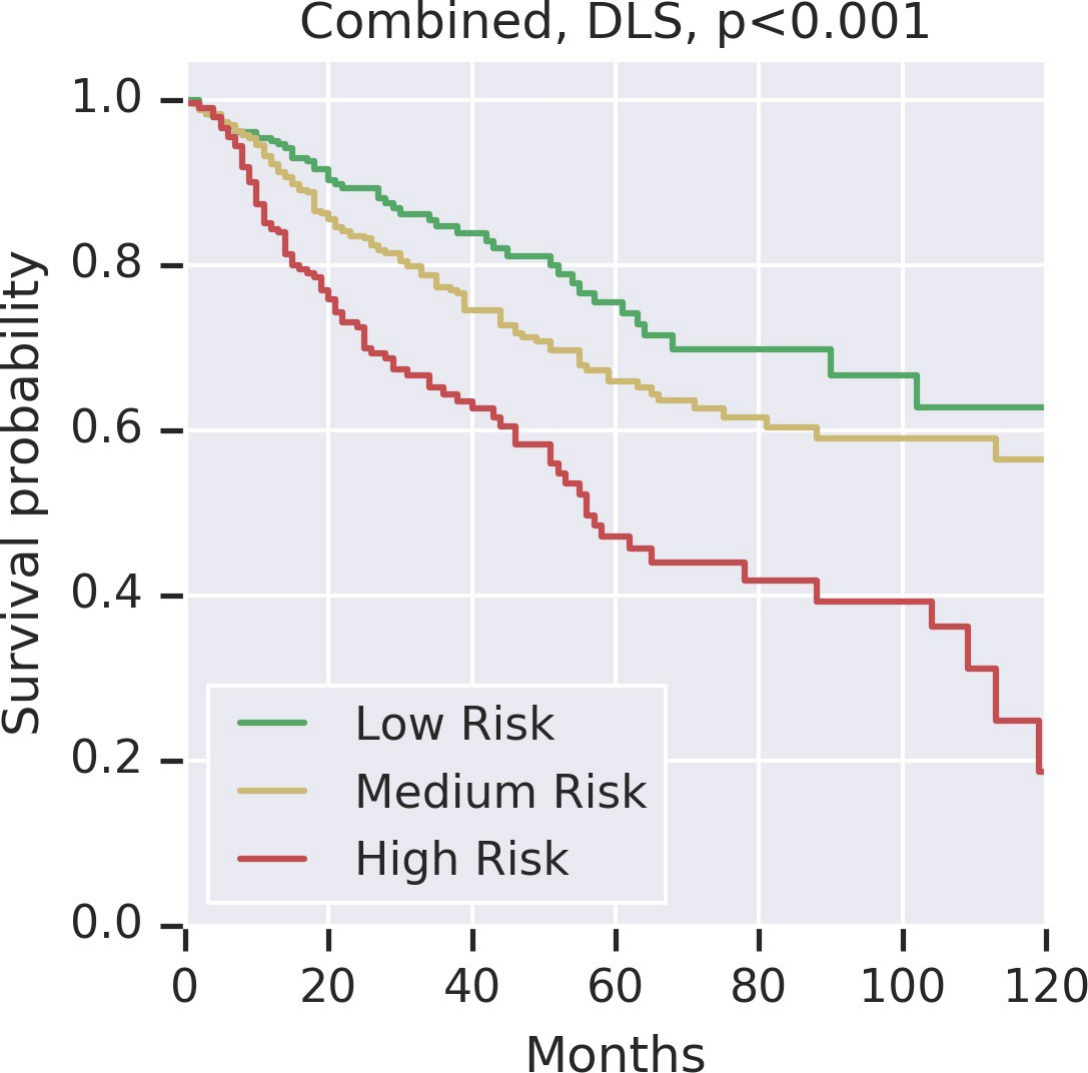
Kaplan Meier curves for DLS risk groups. To define low and high risk groups, cases were binned into risk quartiles using DLS risk scores. Binning was done within each cancer type to ensure that the distribution of cancer types within each risk group was the same. Different colors represent the different risk groups: green for the low risk (0^th^ -25^th^ percentile); yellow for medium risk (25^th^-75^th^ percentile), and red for high risk (75^th^-100^th^ percentile). P-values were calculated using the binary logrank test comparing the low and high risk groups. The Kaplan Meier curve for each individual cancer type is shown in S2 Fig.

The output of the DLS is a continuous risk score that can be used as a feature for survival analysis. To define low and high risk groups, cases were binned into risk quartiles using DLS risk scores. Binning was done within each cancer type to ensure that the distribution of cancer types within each risk group was the same. A logrank test comparison between the Kaplan-Meier (KM) curves for the high and low risk groups yielded p<0.001 (Fig 2).

**Fig 3:**
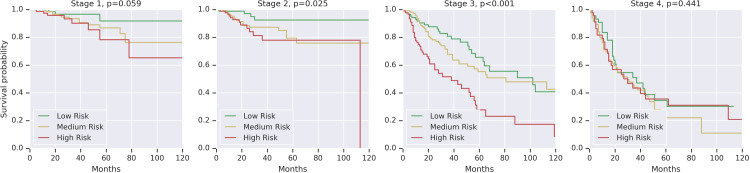
Kaplan Meier curves for DLS risk groups within each cancer stage. To define low and high risk groups, cases were binned into risk quartiles using DLS risk scores. Binning was done within each stage and cancer type. This ensures that for each stage, the distribution of cancer types within each risk group was the same. P-values were calculated using the binary logrank test comparing the low and high risk groups. Unfortunately, there were insufficient cases and events to meaningfully perform this analysis stratified by cancer type in addition to stage.

Given the known prognostic significance of stage, we assessed if the DLS could also sub-stratify patients’ risk within each stage. The resulting Kaplan-Meier curves show that the DLS can further sub-stratify patients into low and high risk groups for stage II (p<0.05) and stage III cancers (p<0.001), but not for stage I or stage IV (Fig 3).

### Multivariable analysis of the DLS and clinical metadata

Next, we used multivariable Cox proportional-hazards regression to evaluate the significance of the DLS as a predictor of disease specific survival after adjusting for available variables: cancer stage, age, and sex. For the combined analysis including all 10 cancer types (i.e. “TCGA studies”), where cancer type was included as an indicator variable, the DLS was a significant predictor of 5-year DSS, with a hazard ratio of 1.48 (p<0.0001). To ensure that age and stage were adequately controlled for across studies, we further fit a combined model with additional interaction terms between the study and stage, and between study and age. In this expanded combined model, the p-value for the DLS remained below 0.001.

In subanalysis of individual cancer types the DLS was significantly associated with disease specific survival for 5 of 10 cancer types, (Table 2; p = 0.0002 to p = 0.0257). Cancer stage was a significant predictor in 7 studies, while age and sex were each significant predictors in only one study each. Univariable analysis is presented in S3 Table.

**Table 2:** Multivariable Cox proportional hazards regression analysis demonstrates association of the Deep Learning System (DLS) with disease-specific survival.

Study	Risk Factor							
	DLS		Age		Male		Stage	
	HR	p	HR	p	HR	p	HR	p
**BLCA**	0.75 [0.45, 1.24]	0.2636	1.27 [0.82, 1.98]	0.2809	1.53 [0.57, 4.11]	0.3939	**2.30 [1.37, 3.86]**	**0.0016**
**BRCA**	**2.86 [1.42, 5.76]**	**0.0034**	1.01 [0.73, 1.40]	0.9412	NaN	NaN	1.72 [0.94, 3.12]	0.0767
**COAD**	**4.03 [1.92, 8.44]**	**0.0002**	0.85 [0.53, 1.38]	0.5086	1.18 [0.38, 3.69]	0.7769	**11.86 [4.18, 33.66]**	**0.0000**
**HNSC**	**2.32 [1.11, 4.88]**	**0.0257**	0.93 [0.63, 1.39]	0.7338	0.91 [0.37, 2.20]	0.8262	**2.26 [1.16, 4.42]**	**0.0171**
**KIRC**	**1.88 [1.23, 2.87]**	**0.0035**	0.99 [0.69, 1.42]	0.9517	0.33 [0.14, 0.77]	**0.0107**	**3.20 [2.02, 5.07]**	**0.0000**
**LIHC**	**2.74 [1.54, 4.86]**	**0.0006**	1.23 [0.84, 1.82]	0.2869	0.99 [0.32, 3.03]	0.9809	**2.31 [1.25, 4.24]**	**0.0072**
**LUAD**	1.35 [0.87, 2.08]	0.1824	0.78 [0.56, 1.10]	0.1546	1.36 [0.58, 3.17]	0.4762	**2.11 [1.50, 2.97]**	**0.0000**
**LUSC**	1.97 [0.90, 4.32]	0.0894	0.83 [0.49, 1.39]	0.4785	1.49 [0.54, 4.14]	0.4404	1.48 [0.91, 2.41]	0.1162
**OV**	1.24 [0.95, 1.63]	0.1157	**1.26 [1.02, 1.55]**	**0.0326**	NaN	NaN	1.45 [0.95, 2.20]	0.0845
**STAD**	1.50 [0.85, 2.62]	0.1602	0.96 [0.69, 1.35]	0.8318	1.94 [0.79, 4.76]	0.1496	**2.19 [1.26, 3.83]**	**0.0058**
**Combined**	**1.48 [1.28, 1.70]**	**<0.0001**	1.07 [0.96, 1.18]	0.2221	1.08 [0.80, 1.48]	0.6063	**2.30 [1.99, 2.66]**	**<0.0001**

Each column header represents one of the input variables for the multivariable analysis, with HR indicating the hazard ratio. For the combined analysis, the study was also included as an indicator variable (coefficients not shown). Univariable analysis is presented in S3 Table, while further analysis adjusting for histologic subtype and grade (where available) are presented in S4 Table. Further analysis restricted to only formalin-fixed paraffin-embedded (FFPE) slides (i.e., discarding frozen slides) are presented in S5 Table.

Although not available for all studies, we also conducted additional multivariable analysis to account for grade and histologic subtype when these data were present in sufficient quantity. These results and the specific histologic subtypes used are summarized in S4 Table. Briefly, the DLS remained a significant predictor of outcome for the same 5 studies as described in Table 2.

Finally, we also performed subanalysis using only the FFPE slides in the test set for evaluation. These results, along with the number of cases for which FFPE slides were available for each cancer type, are summarized in S5 Table. In this analysis using FFPE only slides, the hazard ratio of the DLS remained statistically significant for the combined analysis when analyzed across all studies (p<0.001), and for 3 individual cancer types in subanalysis.

### Measuring the added predictive value of the DLS

The concordance index (or c-index) assesses the goodness-of-fit for a survival model by calculating the probability of the model correctly ordering a (comparable) pair of cases in terms of their survival time [26]. We compared the c-index of Cox-regression models with three different feature sets: (1) “DLS”, consisting of the DLS predictions only; (2) “Baseline”, consisting of stage, age, and sex; and (3) “Baseline+DLS”, consisting of stage, age, sex, and DLS predictions. The c-index results for all cancer types combined and for each cancer type individually are summarized in Table 3. For the DLS model, the c-index for all 10 studies combined (comparisons across cases from different cancer types were excluded) was 61.1 (95% confidence interval (CI) [57.2, 65.1]). Within individual studies, the confidence intervals were too wide to draw meaningful conclusions due to low case volumes. We interpreted the delta in c-index between the “Baseline-only” and the “Baseline+DLS” models as a measure of the added predictive value of the DLS over the baseline variables. For all studies combined, the c-index delta was 3.7 (95% CI [1.0, 6.5]).

**Table 3:** C-index for Cox regression models using DLS and baseline variables as input.

Study	DLS (1)	Baseline (2)	Baseline + DLS (3)	Delta (3–2)
**BLCA**	54.0 [43.3, 64.8]	69.0 [57.4, 80.8]	68.3 [56.0, 80.1]	-0.7 [-4.6, 2.8]
**BRCA**	72.0 [55.5, 87.3]	64.3 [45.6, 78.3]	71.0 [53.8, 85.7]	6.7 [-7.9, 20.6]
**COAD**	70.9 [54.0, 85.4]	80.0 [66.6, 90.9]	91.9 [85.7, 96.6]	**11.9 [3.8, 23.1]**
**HNSC**	58.2 [46.0, 70.0]	49.7 [38.8, 60.4]	64.3 [52.6, 75.0]	**14.5 [5.3, 24.6]**
**KIRC**	71.1 [59.4, 82.5]	85.7 [77.9, 92.4]	85.9 [79.1, 92.2]	0.2 [-3.1, 4.0]
**LIHC**	71.3 [53.8, 88.0]	77.3 [62.3, 88.5]	80.1 [67.6, 91.2]	2.8 [-6.1, 12.6]
**LUAD**	46.3 [32.5, 59.8]	75.4 [65.1, 84.1]	74.8 [64.6, 83.8]	-0.6 [-4.0, 2.7]
**LUSC**	62.1 [47.3, 75.4]	55.7 [42.6, 68.6]	60.3 [46.4, 72.7]	4.6 [-8.7, 15.8]
**OV**	53.9 [45.2, 62.5]	60.3 [52.6, 67.7]	61.3 [53.4, 68.7]	1.0 [-3.4, 5.8]
**STAD**	68.7 [57.8, 78.3]	67.5 [57.5, 77.4]	72.4 [62.3, 81.9]	4.9 [-2.1, 12.0]
**Combined**	61.1 [57.2, 65.1]	66.9 [63.1, 70.8]	70.6 [67.1, 74.2]	**3.7 [1.0, 6.5]**

(1) deep learning system (“DLS-only”), (2) stage, age, sex (“Baseline-only”), or (3) age, stage, sex, and DLS (“Baseline + DLS”). Significant differences based on confidence intervals are highlighted in bold. Corresponding results for 5-year AUC are presented in S6 Table.

In addition to c-index, we also calculated the area under the receiver operating characteristic curve (AUC) for prediction of 5-year disease specific survival. Qualitatively similar results were observed, with the combined analysis showing an AUC improvement of 6.4 (95% CI [2.2, 10.8], S6 Table).

### Understanding the DLS

**Fig 4:**
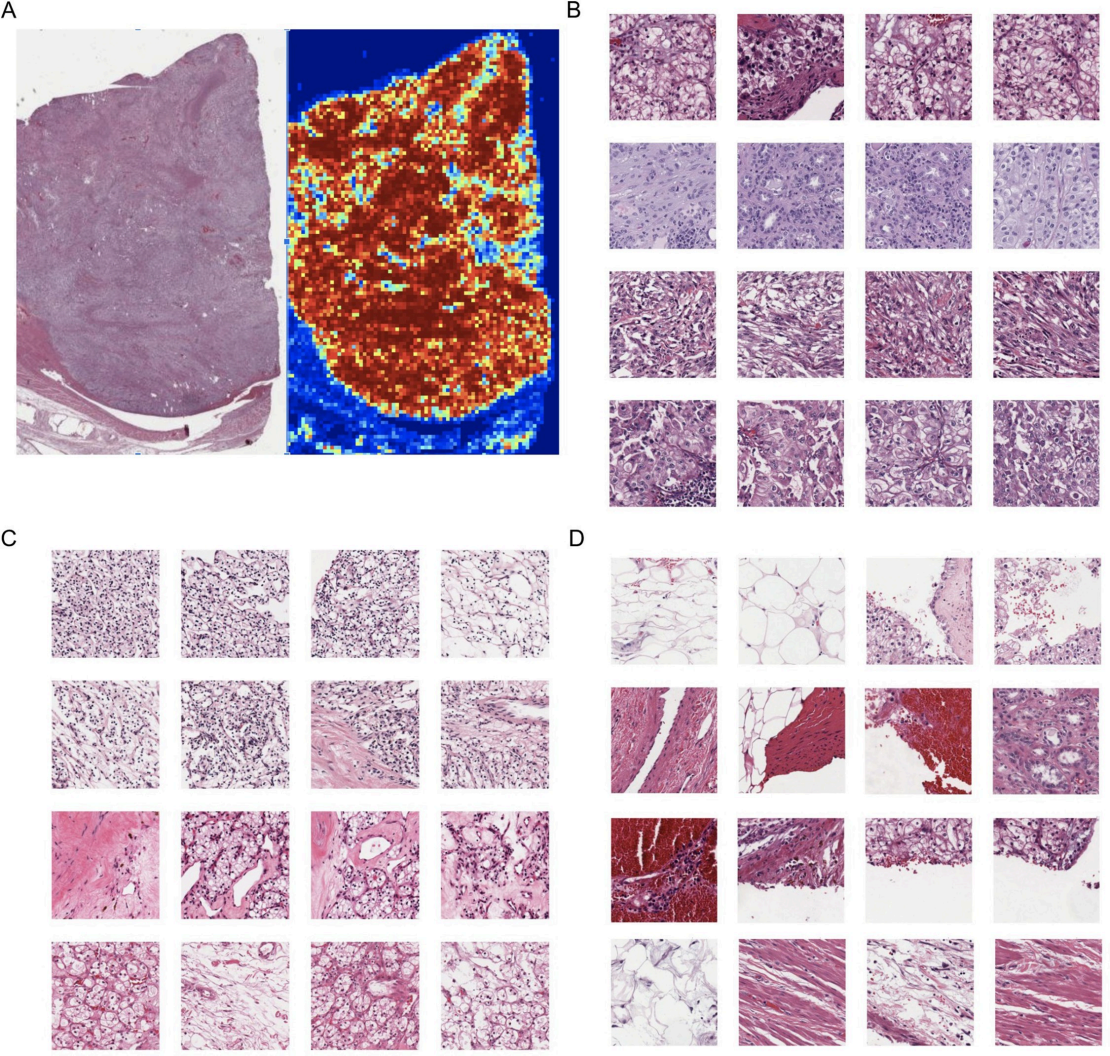
Visualization of image patches influencing survival prediction. (A) Example of WSI kidney renal clear cell carcinoma (KIRC) predicted to be high risk (left), with the DLS-predicted “risk heatmap” on the right; red patches correspond to “high-risk” and blue patches to “low-risk” patch-level predictions (Methods). (B) “Highest-risk” patches from cases predicted to be high-risk. (C) “Lowest-risk” patches from cases predicted to be low-risk. (D) “Lowest-risk” patches from cases predicted to be high-risk. For B, C, and D, patches in the same row are from the same case and each row represents a different case.

To gain initial insights into the DLS, we first computed the correlation of the DLS predictions with the baseline variables of stage, TNM categories, and age. The DLS predictions were not correlated with age in any study, but were correlated with stage and T-category in several cancer types as well as in the combined analysis (S7 Table). Next, we analyzed the regions of each slide that contributed to the overall case classification by extracting the individual patches with the highest and lowest patch-level DLS risk scores for further review. Using KIRC as a representative example with a consistently high-performing DLS model, the patches with the “most confident” predictions for high or low risk tended primarily to contain tumor (Fig 4A–4C), whereas patches with more intermediate prediction values tended to be non-tumor, such as fat, stroma, and fragmented tissue (Fig 4D). In this analysis, more detailed associations of histologic features and patch-level risk predictions were not identified. Samples of high and low risk patches corresponding to the other cancer types for which the DLS provided significant prognostic value are provided in S3–S6 Figs.

### Tradeoffs of weak supervision

Predicting patient prognosis in oncology underlies important clinical decisions regarding treatment and monitoring. In this work, we assessed the potential to improve predictions of disease-specific survival using a deep learning system trained without human annotations for known morphological features or regions of interest.

A natural question arises as to the value of developing algorithms to predict prognosis exclusively from machine learned features, versus leveraging region-level annotations for known features such as tumor grade, nuclear pleomorphism, tumor-infiltrating lymphocytes, or mitotic figures among others. One straightforward advantage is to avoid the cost, tediousness, and difficulties associated with region-level annotations. Furthermore, the relatively unbiased nature of these weakly supervised models potentially enables the learning of previously unknown or unappreciated prognostic features. The primary disadvantage, on the other hand, was the increased number of cases required to train accurate models given that there was only a single case-level training label for each image, such as survival or disease progression. To place the difficulty of this problem in context, these labels correspond to 10^9^ pixels per image, often with several images per case, making for significantly weaker supervision than in typical image prediction tasks that deal with images sized 10^5^−10^6^ pixels. In addition, cancer survival prediction is by nature limited to several orders of magnitude less data than typical image classification problems (e.g. 10^5^−10^6^ images for ImageNet versus 10^2^−10^3^ images here).

The DLS presented in this work learned morphologic features that were predictive of disease-specific survival in multiple cancer types. While we did not identify any clear trends or confounders specific to the cancer types for which the models performed best, future work to better understand the effects of sample size, image-specific variables, and disease-specific variables on clinical predictions from WSIs will be important for the field. Our solution for weak supervision involves a neural network architecture that randomly samples multiple tissue-containing patches for each case at training time. This sampling approach has three main advantages. First, it provides a high probability of seeing patches containing informative features in each training iteration, and even more so across training iterations. Second, assuming each case contains more than one informative image patch, it substantially expands the effective dataset size by increasing the diversity of examples. Third, even uninformative patches have a regularization effect on the training. A similar approach has been explored [18] though only for tissue microarrays of a single cancer type and using image features from a frozen model that was trained on ImageNet. We have provided a more comprehensive analysis than prior work by developing and validating our DLS models across multiple cancer types on WSIs without region of interest annotations.

### Evaluation of learned features

In our study, the fact that the DLS output remained significantly associated with disease specific survival even after adjusting for age and cancer stage suggests that the DLS learned prognostic morphologic features that were independent from these baseline variables. In an effort to better understand some of the learned features, we applied the DLS to every image patch on each slide to obtain “patch-level prognosis estimates” across the entire image. In this analysis, the most confident prognostic regions were comprised primarily of tumor with minimal intervening stroma or other obvious histological structures. While other machine learning efforts have identified prognostic significance for non-tumor elements [17,32], our observations suggest that at least for our specific models, the morphologic features of the tumor appear to be more relevant than non-tumor regions. However, elucidating the morphological features that the DLS learned to help distinguish between high risk and low risk cases remains an exciting but challenging topic for future efforts, and one that will likely require identification of unique features for different tumor types. One intriguing hypothesis is that DLS-learned features may correspond to previously unappreciated representations of tumor biology in the histology, and that underlying biological pathways or molecular mechanisms may be further elucidated via focused evaluation of regions highlighted by the DLS.

### Limitations

Though we have presented promising results for a challenging deep learning problem, there are several notable limitations to our study. First, despite leveraging data across 10 cancer types from the biggest public dataset available (TCGA), each cancer type’s test dataset contained fewer than 250 cases and fewer than 100 disease specific survival events, resulting in wide confidence intervals that limit statistical conclusions (and highlight the importance of reporting model performance confidence intervals when publishing in this field). As such, this work represents a proof-of-concept study to refine methods and to better understand the feasibility of weakly supervised, direct clinical outcome prediction. While the models did learn prognostic signals, these findings require additional development and validation in larger datasets to further improve predictions and more accurately estimate effect sizes, let alone to demonstrate clinical value. Second, our methods and results are limited to datasets from TCGA, for which there are typically a small number of images per case and tumor purity in each image is high [14]. Thus it remains to be seen if the random “patch-sampling” approach described here will be effective in real-world clinical settings where tumor purity is more variable, sectioning protocols may differ, and many slides are typically available for each case. Additionally, while the possible confounding effect of treatment differences between patients were not addressed in these data, all of the patients in these studies were untreated at the time of tissue sampling and the risk stratification on baseline variables shows the expected pattern despite possible differences in treatment. We also note that the DLS was only presented with regions of primary, untreated tumors (as per TCGA inclusion criteria and sampling). While this potentially allowed learning of features associated with the primary tumor such as tumor invasion or grade, the DLS is arguably less likely to have learned features associated with additional specimens such as lymph nodes, margin regions, or metastatic sites. Indeed, the DLS predictions did correlate with the “T” categorization of the TNM staging in the combined analysis, but not with the “N” categorization (S7 Table). Future work using additional slides may be able to further inform risk stratification via learning of additional histological features. Lastly, this work does not specifically incorporate available molecular information from TCGA, which would likely require cancer type-specific molecular analyses and larger datasets.

## Conclusions

In conclusion, we have demonstrated promising results for direct prediction of clinical outcomes in a weakly-supervised setting, without the use of any region-level expert-annotations for training. We hope this work provides useful insights and benchmarks regarding dataset requirements and modeling approaches for survival prediction, especially as it relates to use of the publicly available TCGA data.

## Supporting information

S1 Fig Comparison of loss functions for DLS training. We compared three loss functions for DLS training: 1) censored cross-entropy, 2) Cox partial likelihood, 3) exponential lower bound on concordance index with the TCGA KIRC dataset. For each loss function 3 batch sizes (32, 64, 128) and 4 learning rates (10e-3, 5e-4, 10e-4, 5e-5, 10e-5) were tried. Models were evaluated on the tune split.
(TIFF)

S2 Fig Kaplan Meier curves for DLS risk groups for each cancer type. The top row contains the five cancer types for which the DLS was statistically significantly associated with disease specific survival in multivariable analysis (Table 2).
(TIFF)

S3 Fig Visualization of image patches influencing survival prediction for Breast Invasive Carcinoma (BRCA). High risk patches from highest risk cases (A) and low risk patches from lowest risk cases (B). Patches in the same row are from the same case and each row represents a different case.
(TIFF)

S4 Fig Visualization of image patches influencing survival prediction for Colon Adenocarcinoma (COAD). High risk patches from highest risk cases (A) and low risk patches from lowest risk cases (B). Patches in the same row are from the same case and each row represents a different case.
(TIFF)

S5 Fig Visualization of image patches influencing survival prediction for Head And Neck Squamous Cell Carcinoma (HNSC). High risk patches from highest risk cases (A) and low risk patches from lowest risk cases (B). Patches in the same row are from the same case and each row represents a different case.
(TIFF)

S6 Fig Visualization of image patches influencing survival prediction for Liver Hepatocellular Carcinoma (LIHC). High risk patches from highest risk cases (A) and low risk patches from lowest risk cases (B). Patches in the same row are from the same case and each row represents a different case.
(TIFF)

S1 Table Pathologic stage distribution (number of cases and percentage) for each study. (DOCX)

S2 Table Hyperparameter search space (see Methods for usage). (DOCX)

S3 Table Univariable Cox analysis (see Table 2 for multivariable analysis). (DOCX)

S4 Table Multivariable Cox proportional hazards regression analysis demonstrates association of the Deep Learning System (DLS) with disease-specific survival after adjusting for histologic subtype and grade where available. (DOCX)

S5 Table Multivariable Cox proportional hazards regression analysis demonstrates association of the Deep Learning System (DLS) with disease-specific survival in FFPE-only* cases. (DOCX)

S6 Table AUC for binarized 5-year disease-specific survival (instead of c-index as in Table 3). (DOCX)

S7 Table Correlation of the DLS predictions with clinical variables. (DOCX)

S1 Algorithm Pseudocode for creating the neural network architecture used in this work. For the hyperparameter space, see S2 Table.
(DOCX)
