# Supplementary figures and images for: Deep learning-based survival prediction for multiple cancer types using histopathology images
Source: PLoS One. 2020 Jun 17;15(6):e0233678. doi: 10.1371/journal.pone.0233678 (PMC7299324; doi:10.1371/journal.pone.0233678)

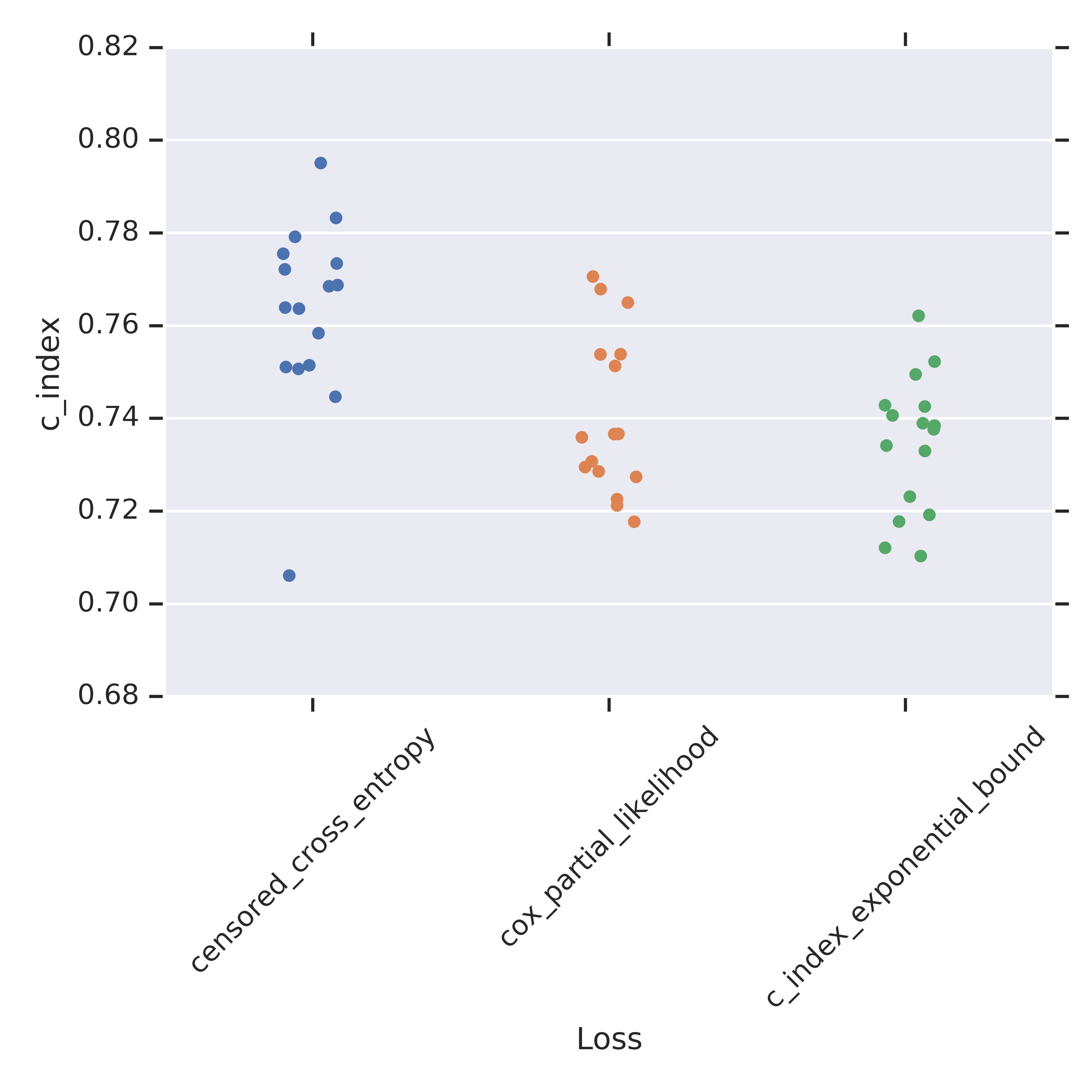

Supplement: S1 Fig — We compared three loss functions for DLS training: 1) censored cross-entropy, 2) Cox partial likelihood, 3) exponential lower bound on concordance index with the TCGA KIRC dataset. For each loss function 3 batch sizes (32, 64, 128) and 4 learning rates (10e-3, 5e-4, 10e-4, 5e-5, 10e-5) were tried. Models were evaluated on the tune split. (TIFF) [file pone.0233678.s001.tiff]

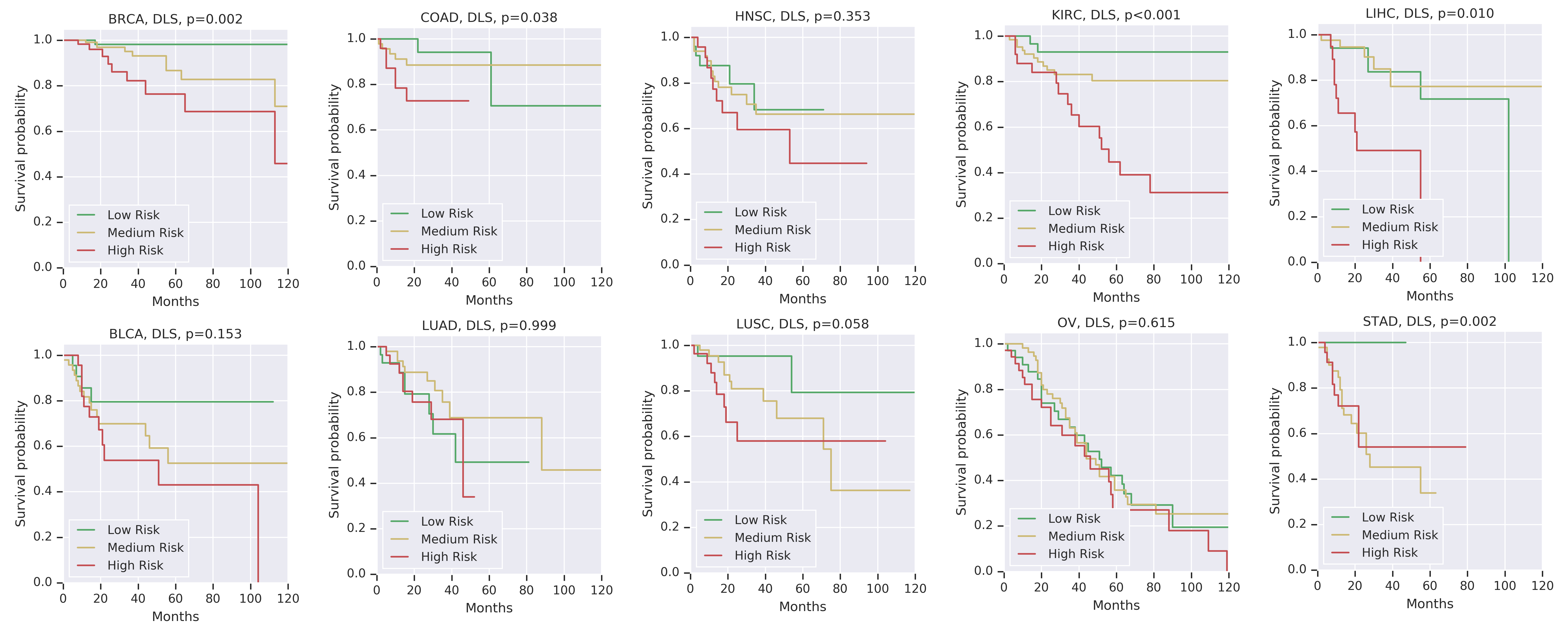

Supplement: S2 Fig — The top row contains the five cancer types for which the DLS was statistically significantly associated with disease specific survival in multivariable analysis (Table 2). (TIFF) [file pone.0233678.s002.tiff]

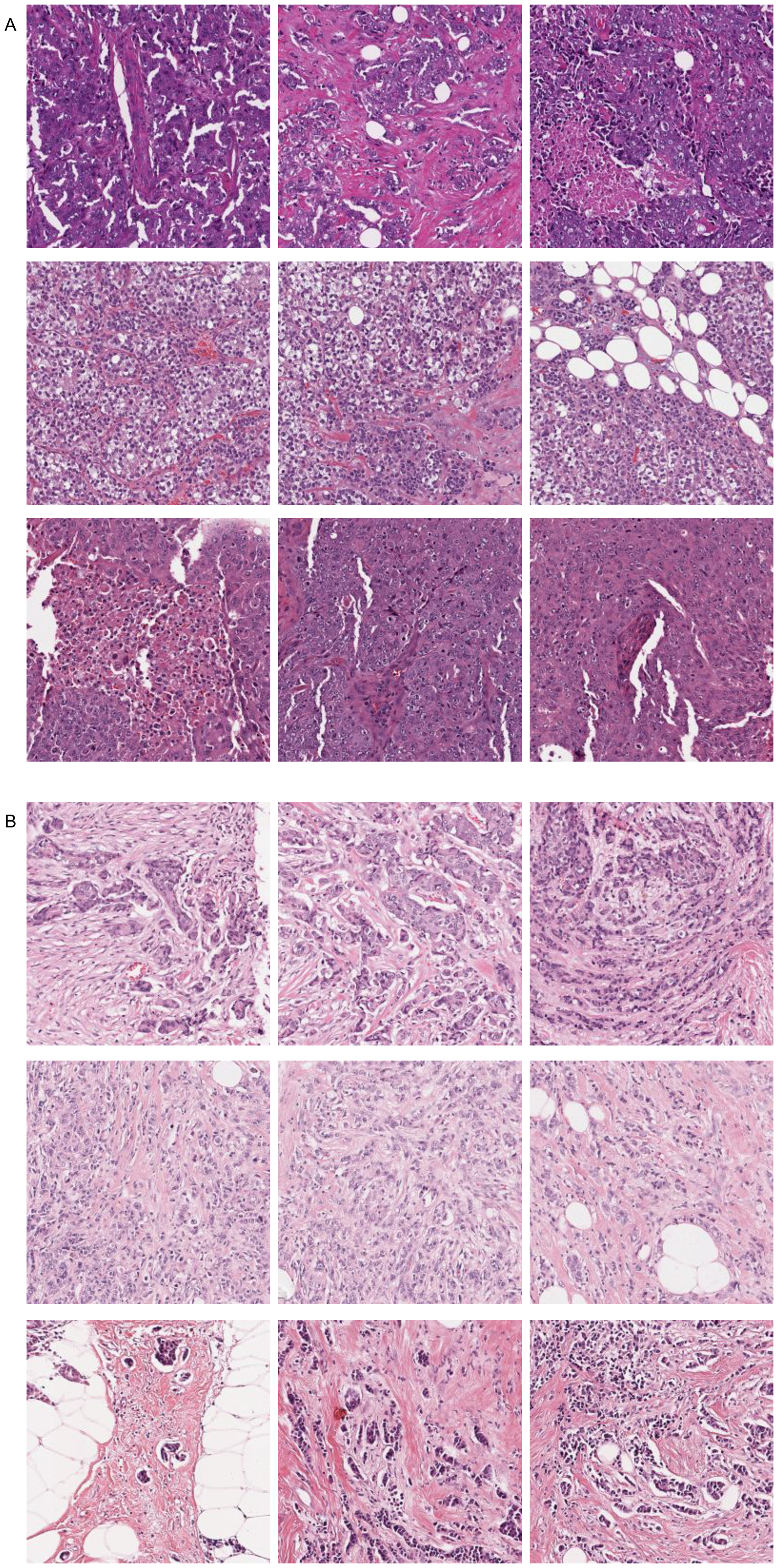

Supplement: S3 Fig — High risk patches from highest risk cases (A) and low risk patches from lowest risk cases (B). Patches in the same row are from the same case and each row represents a different case. (TIFF) [file pone.0233678.s003.tiff]

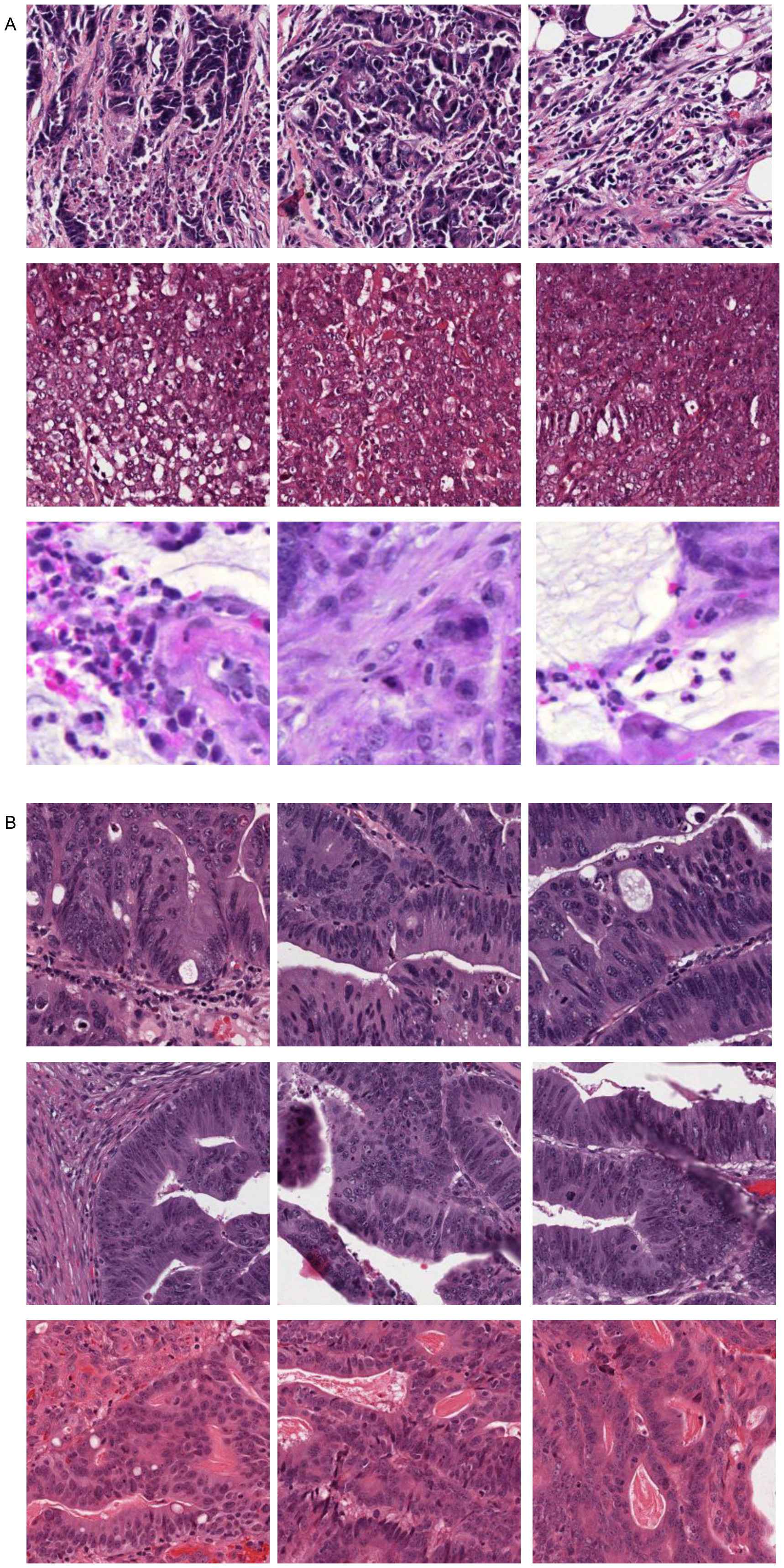

Supplement: S4 Fig — High risk patches from highest risk cases (A) and low risk patches from lowest risk cases (B). Patches in the same row are from the same case and each row represents a different case. (TIFF) [file pone.0233678.s004.tiff]

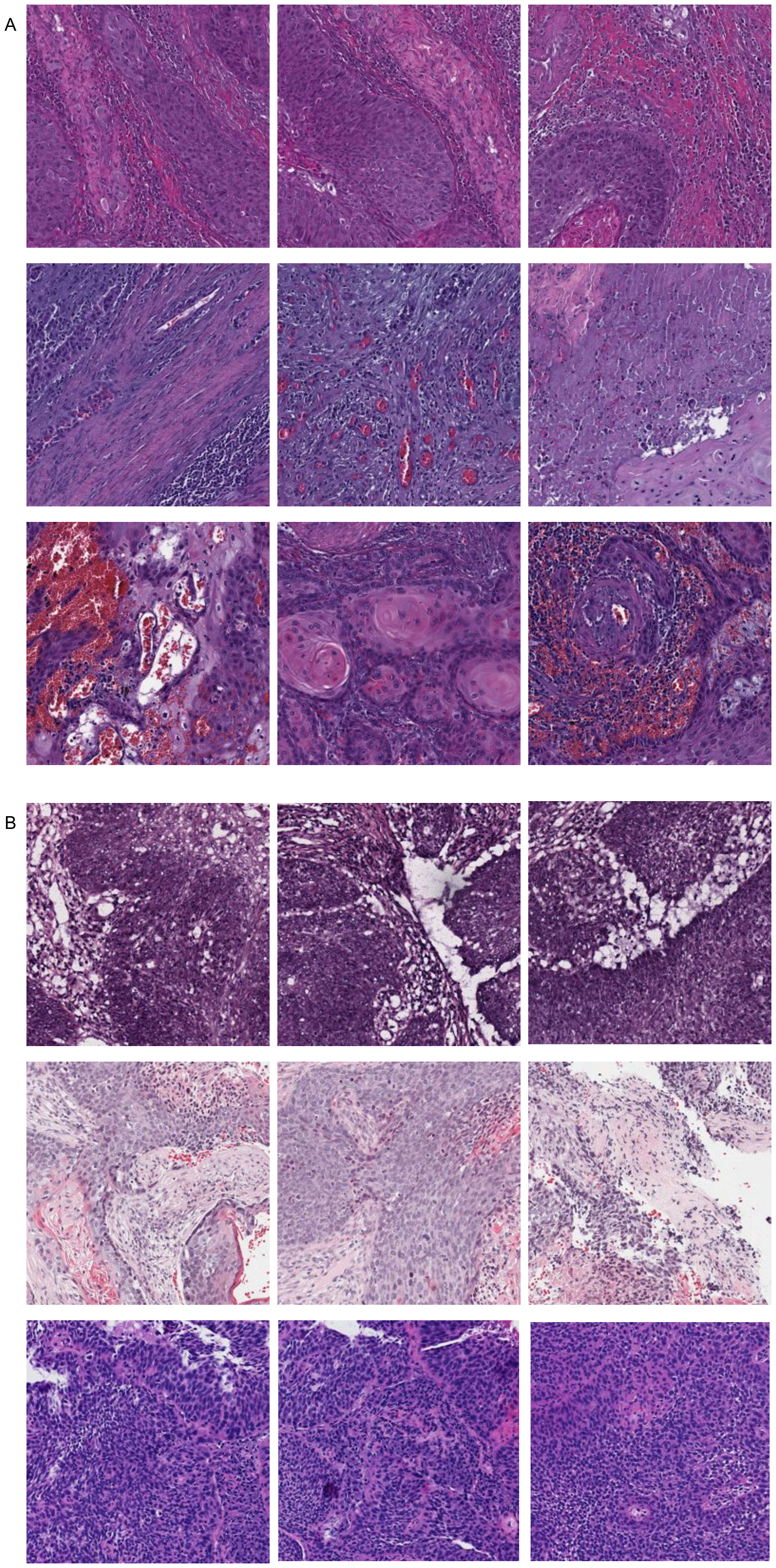

Supplement: S5 Fig — High risk patches from highest risk cases (A) and low risk patches from lowest risk cases (B). Patches in the same row are from the same case and each row represents a different case. (TIFF) [file pone.0233678.s005.tiff]

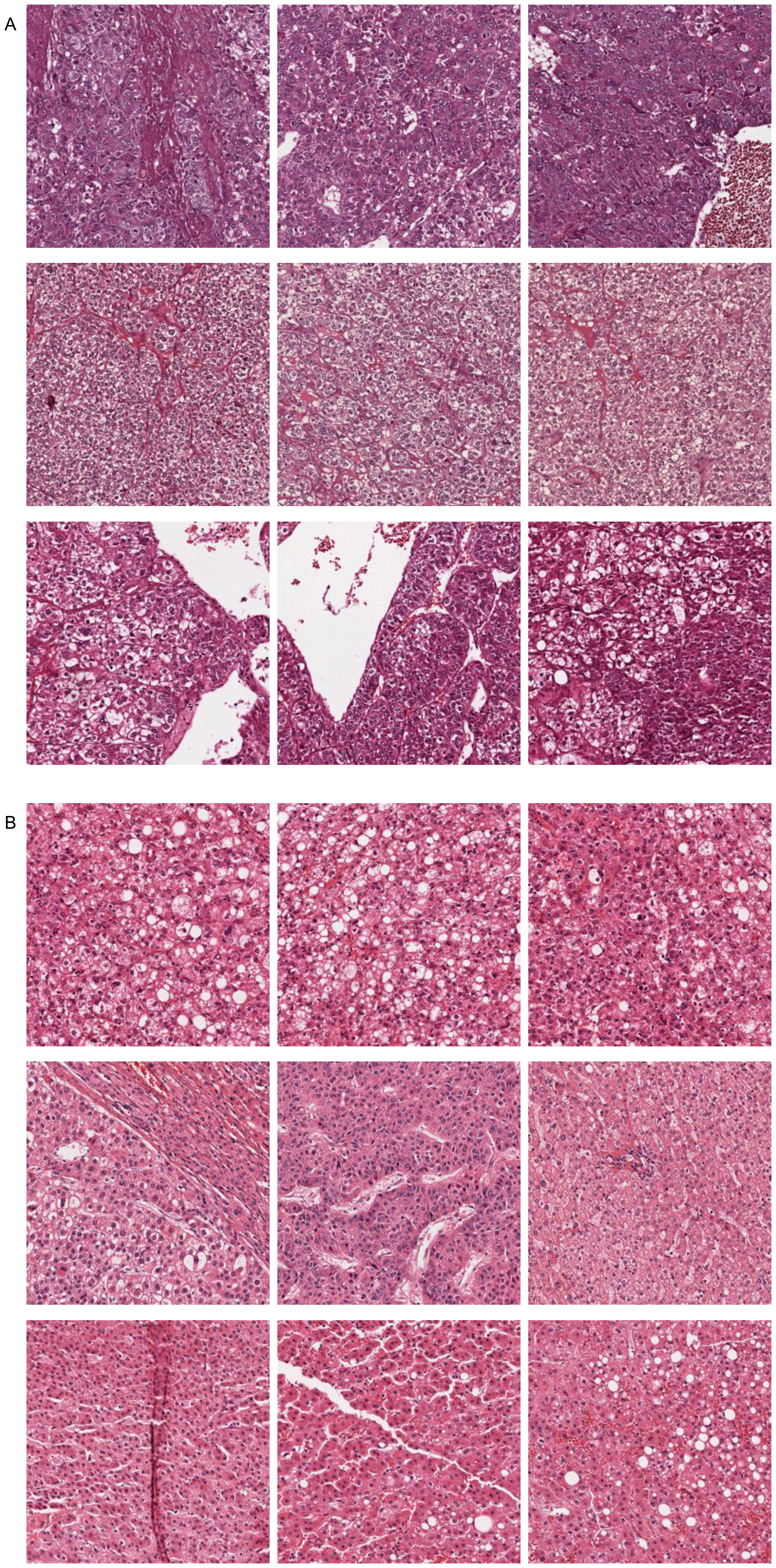

Supplement: S6 Fig — High risk patches from highest risk cases (A) and low risk patches from lowest risk cases (B). Patches in the same row are from the same case and each row represents a different case. (TIFF) [file pone.0233678.s006.tiff]
