# Supplementary material for: Deep learning-based survival prediction for multiple cancer types using histopathology images
Source: PLoS One. 2020 Jun 17;15(6):e0233678. doi: 10.1371/journal.pone.0233678 (PMC7299324; doi:10.1371/journal.pone.0233678)
Supplement: S1 Table — (DOCX) [file pone.0233678.s007.docx]

**S1 Table. Pathologic stage distribution (number of cases and percentage) for each study.**

| **Study** | Dataset split | | | | | | | | | | | |
| --- | --- | --- | --- | --- | --- | --- | --- | --- | --- | --- | --- | --- |
|  | Train | | | | Tune | | | | Test | | | |
|  | I | II | III | IV | I | II | III | IV | I | II | III | IV |
| **BLCA** | 1  (0.5%) | 73  (37.1%) | 62  (31.5%) | 61  (31.0%) | 1  (1.0%) | 26  (26.5%) | 35  (35.7%) | 36  (36.7%) | 0  (0.0%) | 29  (30.2%) | 35  (36.5%) | 32  (33.3%) |
| **BRCA** | 76  (15.6%) | 286  (58.6%) | 118  (24.2%) | 8  (1.6%) | 45  (18.2%) | 145  (58.7%) | 52  (21.1%) | 5  (2.0%) | 53  (21.2%) | 139  (55.6%) | 52  (20.8%) | 6  (2.4%) |
| **COAD** | 33  (15.1%) | 84  (38.5%) | 65  (29.8%) | 36  (16.5%) | 24  (21.8%) | 40  (36.4%) | 33  (30.0%) | 13  (11.8%) | 19  (18.4%) | 44  (42.7%) | 25  (24.3%) | 15  (14.6%) |
| **HNSC** | 11  (5.6%) | 33  (16.8%) | 30  (15.3%) | 122  (62.2%) | 5  (5.1%) | 15  (15.2%) | 21  (21.2%) | 58  (58.6%) | 6  (5.9%) | 15  (14.9%) | 21  (20.8%) | 59  (58.4%) |
| **KIRC** | 132  (50.8%) | 29  (11.2%) | 61  (23.5%) | 38  (14.6%) | 67  (51.5%) | 15  (11.5%) | 27  (20.8%) | 21  (16.2%) | 66  (50.8%) | 12  (9.2%) | 32  (24.6%) | 20  (15.4%) |
| **LIHC** | 79  (47.9%) | 46  (27.9%) | 39  (23.6%) | 1  (0.6%) | 43  (51.8%) | 21  (25.3%) | 19  (22.9%) | 0  (0.0%) | 47  (55.3%) | 13  (15.3%) | 23  (27.1%) | 2  (2.4%) |
| **LUAD** | 117  (50.2%) | 57  (24.5%) | 47  (20.2%) | 12  (5.2%) | 69  (60.0%) | 29  (25.2%) | 12  (10.4%) | 5  (4.3%) | 69  (61.6%) | 25  (22.3%) | 13  (11.6%) | 5  (4.5%) |
| **LUSC** | 113  (51.6%) | 63  (28.8%) | 40  (18.3%) | 3  (1.4%) | 54  (50.0%) | 36  (33.3%) | 17  (15.7%) | 1  (0.9%) | 51  (46.8%) | 42  (38.5%) | 13  (11.9%) | 3  (2.8%) |
| **OV*** | 4  (1.5%) | 19  (7.0%) | 204  (75.0%) | 45  (16.5%) | 3  (2.3%) | 6  (4.5%) | 108  (81.2%) | 16  (12.0%) | 8  (5.8%) | 4  (2.9%) | 102  (74.5%) | 23  (16.8%) |
| **STAD** | 28  (14.1%) | 68  (34.3%) | 83  (41.9%) | 19  (9.6%) | 13  (13.7%) | 26  (27.4%) | 48  (50.5%) | 8  (8.4%) | 12  (12.9%) | 30  (32.3%) | 39  (41.9%) | 12  (12.9%) |
| **Combined** | 594  (24.3%) | 758  (31.0%) | 749  (30.6%) | 345  (14.1%) | 324  (26.6%) | 359  (29.5%) | 372  (30.5%) | 163  (13.4%) | 331  (27.2%) | 353  (29.0%) | 355  (29.2%) | 177  (14.6%) |

*only clinical stage (rather than pathologic stage) was available and used for OV.
