# Supplementary material for: Deep learning-based survival prediction for multiple cancer types using histopathology images
Source: PLoS One. 2020 Jun 17;15(6):e0233678. doi: 10.1371/journal.pone.0233678 (PMC7299324; doi:10.1371/journal.pone.0233678)
Supplement: S2 Table — (DOCX) [file pone.0233678.s008.docx]

**S2 Table. Hyperparameter search space (see Methods for usage).**

| Hyperparameter | Description | Possible values |
| --- | --- | --- |
| Fixation Types | Fixation types for slides for both training and evaluation | FFPE, “FFPE and FROZEN” |
| Patch size | Height and width of each image patch | 256 |
| Patch set size | Number of patches sampled from a case to form a single training example: | 1, 4, 8, 16 |
| Magnification | Image magnification at which the patches are extracted | 20X, 10X, 5X |
| Number of layers | Number of layers used in our MobileNet-based architecture | 4, 8, 12 (i.e., “stride_1_layers” is 0, 1, or 2; see S1 Algorithm) |
| Base depth | Depth of the first convolution layer in the MobilNet CNN; depth grows by a factor of 1.25 for every 2 layers in the network. | 8, 16 |
| L2 regularization weight | Weight of the L2 loss used for regularization | 0.004, 0.0004, 0.00004, 0.000004 |
| Initial Learning rate | Initial learning rate used for the RMSPROP optimizer; decay rate was 0.99 every 20,000 steps. | 0.005, 0.001, 0.0005, 0.0001 |
| Thresholds | Percentile thresholds used for binning time in the censored cross-entropy loss. | [50], [25, 75], [25, 50, 75] |
| Training dataset | Trained on study (cancer-type) specific data, or combined across all cancers | Combined or cancer-specific |

## 
