# Supplementary material for: Deep learning-based survival prediction for multiple cancer types using histopathology images
Source: PLoS One. 2020 Jun 17;15(6):e0233678. doi: 10.1371/journal.pone.0233678 (PMC7299324; doi:10.1371/journal.pone.0233678)
Supplement: S3 Table — (DOCX) [file pone.0233678.s009.docx]

**S3 Table. Univariable Cox analysis (see Table 2 for multivariable analysis).**

| **Study** | **Risk Factor** | | | | | | | |
| --- | --- | --- | --- | --- | --- | --- | --- | --- |
|  | **DLS** | | **Age** | | **Male** | | **Stage** | |
|  | HR | p | HR | p | HR | p | HR | p |
| **BLCA** | 0.81  [0.50, 1.32] | 0.3935 | 1.31  [0.91, 1.90] | 0.1516 | 1.41  [0.54, 3.70] | 0.4801 | **2.48**  **[1.47, 4.20]** | **0.0007** |
| **BRCA** | **3.86**  **[2.15, 6.96]** | **0.0000** | 0.99  [0.71, 1.38] | 0.9509 | NaN | NaN | **2.62**  **[1.54, 4.47]** | **0.0004** |
| **COAD** | **2.09**  **[1.21, 3.63]** | **0.0087** | 0.68  [0.45, 1.03] | 0.0706 | 0.99  [0.33, 2.96] | 0.9827 | **6.66**  **[2.76, 16.09]** | **0.0000** |
| **HNSC** | 1.82  [0.98, 3.40] | 0.0583 | 0.96  [0.66, 1.41] | 0.8406 | 1.07  [0.47, 2.45] | 0.8706 | **1.98**  **[1.08, 3.64]** | **0.0274** |
| **KIRC** | **2.82**  **[1.85, 4.32]** | **0.0000** | 1.14  [0.86, 1.52] | 0.3466 | 0.57  [0.27, 1.22] | 0.1500 | **3.22**  **[2.12, 4.90]** | **0.0000** |
| **LIHC** | **3.26**  **[1.93, 5.53]** | **0.0000** | 0.97  [0.70, 1.35] | 0.8658 | 0.90  [0.35, 2.32] | 0.8298 | **2.60**  **[1.52, 4.42]** | **0.0004** |
| **LUAD** | 1.06  [0.68, 1.65] | 0.7954 | 0.78  [0.56, 1.09] | 0.1464 | 1.35  [0.62, 2.97] | 0.4505 | **2.06**  **[1.48, 2.85]** | **0.0000** |
| **LUSC** | 1.98  [0.99, 3.94] | 0.0517 | 0.93  [0.58, 1.48] | 0.7615 | 1.69  [0.62, 4.62] | 0.3075 | **1.68**  **[1.05, 2.68]** | **0.0291** |
| **OV** | 1.19  [0.90, 1.55] | 0.2168 | 1.22  [0.98, 1.50] | 0.0703 | NaN | NaN | 1.35  [0.90, 2.03] | 0.1446 |
| **STAD** | 1.74  [1.05, 2.90] | 0.0329 | 0.89  [0.64, 1.25] | 0.5090 | 1.86  [0.78, 4.47] | 0.1636 | **2.26**  **[1.34, 3.82]** | **0.0023** |

## 
