# Supplementary material for: Deep learning-based survival prediction for multiple cancer types using histopathology images
Source: PLoS One. 2020 Jun 17;15(6):e0233678. doi: 10.1371/journal.pone.0233678 (PMC7299324; doi:10.1371/journal.pone.0233678)
Supplement: S4 Table — (DOCX) [file pone.0233678.s010.docx]

**S4 Table. Multivariable Cox proportional hazards regression analysis demonstrates association of the deep learning system (DLS) with disease-specific survival after adjusting for histologic subtype and grade where available.**

| **Study** | **DLS in multivariable model without subtype/stage** | | | **DLS in multivariable model with subtype/stage** | | |
| --- | --- | --- | --- | --- | --- | --- |
|  | HR | p | Baseline variables adjusted for | HR | p | Baseline variables adjusted for |
| **BLCA** | 0.75  [0.45, 1.24] | 0.2636 | Age, Gender, Stage | 0.79  [0.46, 1.34] | 0.3780 | Age, Gender, Stage, Grade |
| **BRCA** | **2.86**  **[1.42, 5.76]** | **0.0034** | Age, Gender, Stage | **2.53**  **[1.24, 5.16]** | **0.0110** | Age, Gender, Stage, Subtype (Ductal, Lobular, Other) |
| **COAD** | **4.03**  **[1.92, 8.44]** | **0.0002** | Age, Gender, Stage | **3.98**  **[1.86, 8.55]** | **0.0003** | Age, Gender, Stage, Subtype (Adenocarcinoma, Mucinous adenocarcinoma) |
| **HNSC** | **2.32**  **[1.11, 4.88]** | **0.0257** | Age, Gender, Stage | **2.38**  **[1.17, 4.86]** | **0.0170** | Age, Gender, Stage, Grade |
| **KIRC** | **1.88**  **[1.23, 2.87]** | **0.0035** | Age, Gender, Stage | **1.79**  **[1.14, 2.82]** | **0.0115** | Age, Gender, Stage, Grade |
| **LIHC** | **2.74**  **[1.54, 4.86]** | **0.0006** | Age, Gender, Stage | **2.79**  **[1.56, 4.98]** | **0.0005** | Age, Gender, Stage, Grade |
| **LUAD** | 1.35  [0.87, 2.08] | 0.1824 | Age, Gender, Stage | 1.44  [0.91, 2.26] | 0.1160 | Age, Gender, Stage, Subtype (Adenocarcinoma NOS, Mixed subtype, Other) |
| **LUSC** | 1.97  [0.90, 4.32] | 0.0894 | Age, Gender, Stage | 1.97  [0.90, 4.32] | 0.0894 | Age, Gender, Stage |
| **OV** | 1.24  [0.95, 1.63] | 0.1157 | Age, Gender, Stage | 1.23  [0.93, 1.61] | 0.1404 | Age, Gender, Stage, Grade |
| **STAD** | 1.50  [0.85, 2.62] | 0.1602 | Age, Gender, Stage | 1.65  [0.91, 3.02] | 0.1013 | Age, Gender, Stage, Grade, Subtype (Diffuse, Intestinal) |
