# Supplementary material for: Deep learning-based survival prediction for multiple cancer types using histopathology images
Source: PLoS One. 2020 Jun 17;15(6):e0233678. doi: 10.1371/journal.pone.0233678 (PMC7299324; doi:10.1371/journal.pone.0233678)
Supplement: S5 Table — (DOCX) [file pone.0233678.s011.docx]

**S5 Table. Multivariable Cox proportional hazards regression analysis demonstrates association of the deep learning system (DLS) with disease-specific survival in FFPE-only* cases.**

| **Study** | **Number of cases (excludes frozen slides)** | **Risk Factor** | | | | | | | |
| --- | --- | --- | --- | --- | --- | --- | --- | --- | --- |
|  |  | **DLS** | | **Age** | | **Male** | | **Stage** | |
|  |  | HR | p | HR | p | HR | p | HR | p |
| **BLCA** | 91 | 0.70  [0.37, 1.31] | 0.262 | 1.23  [0.78, 1.95] | 0.3657 | 1.45  [0.54, 3.91] | 0.461 | **2.27**  **[1.33, 3.87]** | **0.003** |
| BRCA | 244 | 2.02  [0.95, 4.30] | 0.068 | 0.99  [0.70, 1.41] | 0.9754 | NaN | NaN | **2.70**  **[1.51, 4.84]** | **0.0009** |
| **COAD** | 100 | **5.35**  **[1.78, 16.08]** | **0.002** | 1.02  [0.61, 1.72] | 0.9288 | 0.93  [0.26, 3.29] | 0.911 | **10.79**  **[3.32, 35.08]** | **0.0001** |
| HNSC | 95 | 0.98  [0.52, 1.84] | 0.948 | 0.92  [0.60, 1.40] | 0.7013 | 0.92  [0.36, 2.32] | 0.856 | **2.22**  **[1.11, 4.44]** | **0.0247** |
| **KIRC** | 128 | **1.50**  **[1.04, 2.16]** | **0.031** | 1.00  [0.67, 1.47] | 0.9835 | 0.47  [0.21, 1.06] | 0.068 | **3.34**  **[2.11, 5.30]** | **0.0000** |
| **LIHC** | 82 | **3.52**  **[1.79, 6.91]** | **0.0003** | 1.31  [0.87, 1.97] | 0.1998 | 0.91  [0.28, 2.94] | 0.880 | **2.22**  **[1.23, 4.02]** | **0.0084** |
| **LUAD** | 104 | 1.48  [0.94, 2.34] | 0.090 | 0.88  [0.61, 1.26] | 0.4741 | 1.29  [0.55, 3.06] | 0.558 | **2.05**  **[1.47, 2.86]** | **0.0000** |
| **LUSC** | 101 | 1.30  [0.65, 2.60] | 0.465 | 0.98  [0.58, 1.67] | 0.9504 | 1.34  [0.48, 3.79] | 0.575 | 1.59  [0.96, 2.66] | 0.074 |
| **STAD** | 84 | 0.78  [0.41, 1.47] | 0.443 | 0.95  [0.68, 1.33] | 0.7663 | 1.87  [0.77, 4.55] | 0.170 | **2.40**  **[1.25, 4.59]** | **0.0082** |
| **Combined** | 1053 | **1.42**  **[1.21, 1.66]** | **0.0000** | 1.09  [0.98, 1.22] | 0.1289 | 1.22  [0.93, 1.61] | 0.158 | **2.20**  **[1.90, 2.53]** | **0.0000** |

*FFPE indicates formalin-fixed paraffin-embedded, “FFPE-only” indicates that images specified by TCGA as “frozen” specimens/slides have been excluded.
