# Supplementary material for: Deep learning-based survival prediction for multiple cancer types using histopathology images
Source: PLoS One. 2020 Jun 17;15(6):e0233678. doi: 10.1371/journal.pone.0233678 (PMC7299324; doi:10.1371/journal.pone.0233678)
Supplement: S6 Table — (DOCX) [file pone.0233678.s012.docx]

**S6 Table. AUC for binarized 5-year disease-specific survival (instead of c-index as in Table 3).**

| **Study** | **DLS (1)** | **Baseline (2)** | **Baseline + DLS (3)** | **Delta (3 -2)** |
| --- | --- | --- | --- | --- |
| **BLCA** | 53.1 [31.9, 73.8] | 68.3 [46.2, 87.7] | 69.9 [50.3, 87.3] | 1.6 [-9.5, 14.2] |
| **BRCA** | 74.0 [58.7, 87.6] | 58.4 [37.3, 75.7] | 70.0 [51.3, 85.6] | **11.6 [-1.5, 25.0]** |
| **COAD** | 87.5 [66.7, 100.0] | 72.9 [43.3, 97.3] | 94.8 [80.0, 100.0] | **21.9 [2.7, 41.7]** |
| **HNSC** | 53.5 [33.5, 73.8] | 39.9 [15.4, 66.7] | 60.1 [38.2, 82.5] | 20.2 [-1.3, 44.8] |
| **KIRC** | 72.8 [59.4, 85.1] | 84.5 [73.4, 94.1] | 87.7 [77.1, 96.3] | 3.2 [-1.3, 8.4] |
| **LIHC** | 77.6 [56.1, 93.8] | 65.6 [41.7, 87.4] | 74.1 [52.4, 91.8] | 8.5 [-9.4, 27.0] |
| **LUAD** | 50.9 [29.6, 70.8] | 66.3 [39.4, 89.6] | 67.1 [40.8, 90.6] | 0.9 [-2.9, 6.2] |
| **LUSC** | 61.1 [36.7, 82.0] | 65.6 [45.5, 84.0] | 76.1 [56.0, 92.9] | 10.5 [-8.1, 30.5] |
| **OV** | 59.9 [47.6, 72.6] | 56.6 [43.1, 68.6] | 60.5 [46.3, 72.2] | 4.0 [-3.3, 11.3] |
| **STAD*** | 28.0 [0.0, 65.5] | 64.0 [4.1, 100.0] | 51.3 [4.1, 100.0] | -12.7 [-42.4, 0.0] |
| **Combined** | 64.3 [58.0, 70.3] | 63.7 [57.1, 70.8] | 70.1 [63.8, 76.8] | **6.4 [2.2, 10.8]** |

*In STAD, only 3 cases had at least 5 years of follow-up and survived for at least 5 years.
