# Supplementary material for: Deep learning-based survival prediction for multiple cancer types using histopathology images
Source: PLoS One. 2020 Jun 17;15(6):e0233678. doi: 10.1371/journal.pone.0233678 (PMC7299324; doi:10.1371/journal.pone.0233678)
Supplement: S7 Table — (DOCX) [file pone.0233678.s013.docx]

**S7 Table. Correlation of the DLS predictions with clinical variables.**

|  | **Stage** | **T** | **N** | **M** | **Age** |
| --- | --- | --- | --- | --- | --- |
| **BLCA** | 7.54 (0.465) | **15.82 (0.124)** | 6.69 (0.517) | 15.50 (0.132) | 6.79 (0.511) |
| **BRCA** | **24.39 (0.000)** | **31.00 (0.000)** | **12.23 (0.053)** | **12.16 (0.055)** | -7.39 (0.244) |
| **COAD** | 0.39 (0.969) | -0.73 (0.942) | 3.80 (0.703) | 2.90 (0.771) | 3.16 (0.751) |
| **HNSC** | 6.42 (0.524) | 14.92 (0.136) | -1.90 (0.851) | **-1.54 (0.878)** | -0.94 (0.926) |
| **KIRC** | **29.07 (0.001)** | **27.77 (0.001)** | -13.34 (0.130) | 5.39 (0.542) | 14.11 (0.109) |
| **LIHC** | **23.01 (0.034)** | **24.85 (0.022)** | -11.14 (0.310) | -5.20 (0.636) | -12.73 (0.246) |
| **LUAD** | -3.30 (0.730) | 7.41 (0.438) | -4.82 (0.614) | 17.33 (0.068) | 8.50 (0.373) |
| **LUSC** | 8.91 (0.357) | 17.99 (0.061) | 3.75 (0.699) | -5.09 (0.599) | 20.55 (0.032) |
| **OV** | -5.81 (0.500) | N/A | N/A | N/A | N/A |
| **STAD** | **22.21 (0.032)** | -2.08 (0.843) | 8.26 (0.431) | **27.00 (0.009)** | -5.93 (0.572) |
| **Combined** | **15.55 (0.000)** | **18.95 (0.000)** | 1.59 (0.602) | 5.46 (0.073) | 5.34 (0.079) |

Spearman’s rank correlation and p-values (with significant values in bold) for correlation of each variable with DLS predictions across cancer types. The Spearman’s rank correlation coefficient was used to account for different numerical scales of each variable.
