# Supplementary material for: Deep learning-based survival prediction for multiple cancer types using histopathology images
Source: PLoS One. 2020 Jun 17;15(6):e0233678. doi: 10.1371/journal.pone.0233678 (PMC7299324; doi:10.1371/journal.pone.0233678)
Supplement: S1 Algorithm — For the hyperparameter space, see S2 Table. (DOCX) [file pone.0233678.s014.docx]

**S1 Algorithm. Pseudocode for creating the neural network architecture used in this work.** For the hyperparameter space, see S2 Table.

| // Depth of the first convolution layer (tuned)  base_depth := 16  // Rate of depth growth with every stride 2 layer (fixed)  depth_growth := 1.25  // Num stride 2 layers (fixed)  stride_2_layers := 4  // Num stride 1 layers per stride 2 layer (tuned)  stride_1_layers := 1  // Size of convolution kernel (fixed)  kernel_size := 3  features = Conv2D(images, base_depth, kernel_size, stride=1)  **for** i := 1 to stride_2_layers **do**  [depth](https://cs.corp.google.com/piper///depot/google3/medical/pathology/tensorflow/components/network/mobilenet.py?l=189&gs=kythe%253A%252F%252Fgoogle3%253Flang%253Dpython%253Fpath%253Dmedical%252Fpathology%252Ftensorflow%252Fcomponents%252Fnetwork%252Fmobilenet.py%2523module.get_mobilenet_conv_defs.depth&gsn=depth&ct=xref_usages) = int([base_depth](https://cs.corp.google.com/piper///depot/google3/medical/pathology/tensorflow/components/network/mobilenet.py?l=159&ct=xref_jump_to_def&gsn=base_depth&rcl=300225872) * [depth_growth](https://cs.corp.google.com/piper///depot/google3/medical/pathology/tensorflow/components/network/mobilenet.py?l=160&ct=xref_jump_to_def&gsn=depth_growth&rcl=300225872) ** [i](https://cs.corp.google.com/piper///depot/google3/medical/pathology/tensorflow/components/network/mobilenet.py?l=188&ct=xref_jump_to_def&gsn=i&rcl=300225872))  // [DepthwiseSeparableConv](https://cs.corp.google.com/piper///depot/google3/medical/pathology/tensorflow/components/network/mobilenet.py?l=32&ct=xref_jump_to_def&gsn=DepthSepConvDef&rcl=300225872)2D is available as tf.keras.layers.SeparableConv2D  features = [DepthwiseSeparableConv](https://cs.corp.google.com/piper///depot/google3/medical/pathology/tensorflow/components/network/mobilenet.py?l=32&ct=xref_jump_to_def&gsn=DepthSepConvDef&rcl=300225872)2D(features, depth, kernel_size, stride=2)  **for**j := 1 to [stride_1_layers](https://cs.corp.google.com/piper///depot/google3/medical/pathology/tensorflow/components/network/mobilenet.py?l=162&ct=xref_jump_to_def&gsn=num_stride_1_layers&rcl=300225872) **do**  features = [DepthwiseSeparableConv](https://cs.corp.google.com/piper///depot/google3/medical/pathology/tensorflow/components/network/mobilenet.py?l=32&ct=xref_jump_to_def&gsn=DepthSepConvDef&rcl=300225872)2D(features, depth, kernel_size, stride=1)  features =AveragePooling2D(features) |
| --- |
